# Biobank Digitalization: From Data Acquisition to Efficient Use

**DOI:** 10.3390/biology13120957

**Published:** 2024-11-22

**Authors:** Anastasiia S. Bukreeva, Kristina A. Malsagova, Denis V. Petrovskiy, Tatiana V. Butkova, Valeriya I. Nakhod, Vladimir R. Rudnev, Alexander A. Izotov, Anna L. Kaysheva

**Affiliations:** Institute of Biomedical Chemistry, 109028 Moscow, Russia; a.bukreeva42@gmail.com (A.S.B.); petro2017@mail.ru (D.V.P.); t.butkova@gmail.com (T.V.B.); vnakhod88@gmail.com (V.I.N.); v.r.rudnev@gmail.com (V.R.R.); izotov.alexander.ibmc@gmail.com (A.A.I.); kaysheva1@gmail.com (A.L.K.)

**Keywords:** biobanking, digital biobanks, biosample, personalized medicine, digitalization

## Abstract

Biobanks are involved in a broad range of studies, including both basic and clinical research, so their functions and roles are evolving. Digital biobanks have emerged due to digitalization in this field; however, it also entails an increasing number of ethical and legal issues, in particular those related to the protection of donor data and potential commercial applications. The development of biobanks and the size of stored datasets lay the groundwork for proceeding to digital biobanks that intensely employ artificial intelligence tools. Digital biobanks can simplify the search for and access to biological specimens, thus contributing to the conduction of research and creating new collaborations. They are becoming an increasingly important tool for personalized medicine and an individualized approach to disease treatment, contributing to the elaboration of more accurate diagnostic methods and the development of innovative therapeutic strategies.

## Simple Summary

In recent years, the introduction of a wide range of digital technologies has changed various workflows in the field of biobanking. There are many systems and technologies that can be used in biobanking procedures, medical research and practice. These digital technologies can bring about significant benefits but also a great responsibility. This review discusses the digitalization of biobanking processes, issues related to the benefits of digital biobanks, as well as the challenges that hinder their development.

## 1. Introduction

Biobanks are an integral component of large-scale medical research projects. Their major objective is to ensure broad uninterrupted access to biomaterial collections for researchers. It is crucial to provide professional information support or sample annotation (i.e., comprehensive clinical and laboratory data about a patient) [1].

The biobanking activity has recently been segregated into a separate discipline, aiming to empower a broad range of research areas and improve the quality of science and healthcare [2]. Precision medicine studies are based on analyzing biological samples characterized by a large amount of data [3,4,5,6]. It is the main premise for developing, implementing, and using such tools as “artificial intelligence” and “big data analytics” in both science and medicine. The high availability of well-characterized high-quality samples is directly related to the pace of the development of various studies that focus on rendering medical aid and improving patients’ quality of life. In turn, it demands that biobanks have increased capacity and ample information capabilities.

Today, the digitalization of biobanks and biospecimen collections is a healthcare trend, combining cutting-edge clinical and fundamental experience with digital technologies [7]. A digital biobank is a unified information system that allows one to control a geographically distributed collection of biospecimens (i.e., enable laboratories and biobanks of individual regions and/or countries to swiftly exchange information and make queries about providing biospecimens for research and development).

Digital biobanks are usually used for detecting genes, biomarkers, gene–environment interplay, and pharmacogenetic associations. They generate knowledge that allows one to manage priorities regarding target drugs and improve the classification of diseases, thus contributing to the development of specialized medical care. The success of these resources is demonstrated by the rapid spread of digital biobanks across the globe; they aggregately collect data on millions of study subjects [8]. For a biobank to function efficiently, one needs to design software and a database and organize such processes as registration, specimen preparation, as well as storage and release of biospecimens to researchers through a digital platform.

The existing digital biobanks most frequently function as an internal information platform and are used for conducting in-house research of companies, laboratories, or research organizations. It is topical to scale up this platform to set up a collaboration with clinical and research organizations, which would have a functional module to handle queries to provide well-characterized high-quality biospecimens and ensure secure data transfers.

This study addresses issues related to the advantages of digital biobanks, the digitalization of operational procedures, and problems hampering the development of biobank digitalization.

## 2. Materials and Methods

**Figure 1:**
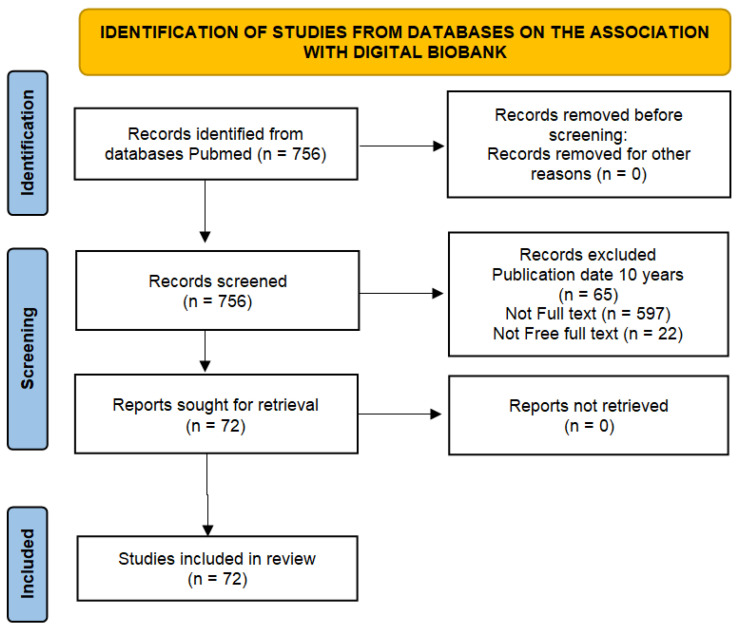
Flow diagram of the review.

An analysis of the literature on research related to the organization of biobank digitalization was carried out in September 2024 using the PubMed database. Our search criteria used the words “digital biobank”. A flowchart of the search strategy is illustrated in Figure 1.

The keyword search yielded 756 publications. Applying search depth filters (10 years), “full text”, and “free full text” reduced the number of publications for further analysis (n = 72). Articles without complete texts were excluded from the analysis due to insufficient information. Consequently, 72 articles with fully accessible text were selected for final review. The exclusion criterion included publications associated with studies involving collections of non-human biological material.

## 3. Biobanking and Digitalization

**Figure 2:**
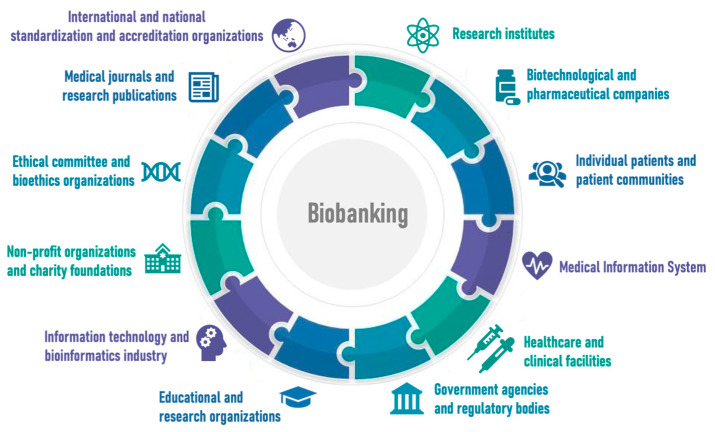
Biobanks and communication of different directions.

Biobanks play a crucial role in coordination and communication within different areas. They are engaged in collaboration with research institutions, clinical centers, and the pharmaceutical industry, as well as other stakeholders. Biobanks help provide access to biological specimens and associated data for research, clinical trials and the development of novel methods for disease diagnosis and treatment [9]. They also facilitate knowledge and expertise sharing between different institutions and scientists, thus accelerating scientific progress and improving research outcomes. Furthermore, biobanks significantly contribute to the training and education of biomedical science professionals and help disseminate best practices and working methods (Figure 2).

Today, digitalization is commonly used in various fields of modern science. Since biobanks are increasingly becoming a component of the research infrastructure, the digitalization of standard operating procedures for biobanks is an important aspect of biobanking.

Over the past few years, stricter requirements have been posed to biospecimen quality: the reliability and quality of the preanalytical stage (biomaterial processing and storage) plays a pivotal role in the reproducibility of the results of the studies [10]. In particular, biospecimen stability and quality are significantly affected by the time interval between the instant of biomaterial collection and analysis. Hence, it follows that it is extremely important to obtain accurate information about the time when the biomaterial was collected [11]. In this case, digital solutions will allow for the integration of digital data sources and the automation of technical processes (time of biospecimen retrieval from cryostorage, processes used to preliminarily prepare them for analysis).

Biobanks can improve their market competitiveness by launching automated control and accounting for the overall lifecycle of biospecimens, which involves:

- Biomaterial collection;
- Shelf life;
- Pre-analytical processing of a biospecimen;
- Freeze–thaw cycles;
- Biospecimen analysis (analytical stage);
- Disposal [10].

Moreover, the volume of data generated by biobanking activities also necessitates digitalization. Digital tools specifically designed for performing biobanking operations (virtual databases and software) facilitate managing large data sets and allow tracking of how biospecimens are transferred from the collection site to a laboratory and/or cryopreservation facility. Hence, digitalization will make it possible to:

- Significantly simplify biobank management by increasing the speed and reliability of data processing;
- Digitization of information will allow researchers to quickly find and access specific biospecimens;
- Digitization of information will reduce errors that are inevitable when using manual data entry and processing;
- Improve data security (advanced security features and resource access control reduce the risk of unauthorized access to data and adequately protect confidentiality) [12].

Furthermore, digitalization endows biobanking with certain advantages: fast-tracking of biospecimen routing, high data security and operational efficiency, ensuring information sharing, networking and collaboration among biobanks.

### 3.1. Operating Procedures

**Figure 3:**
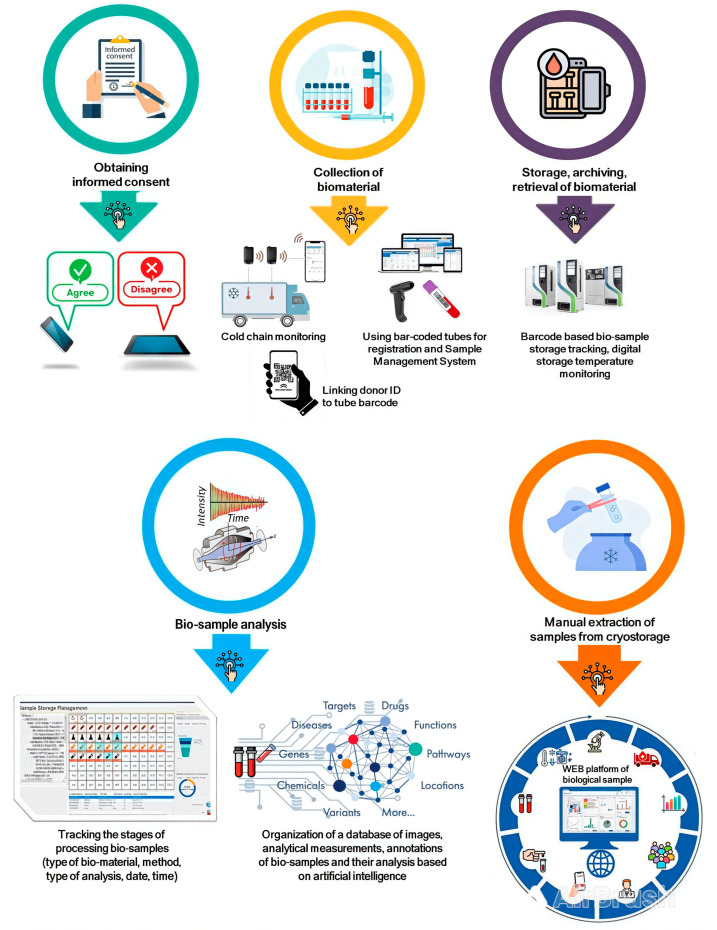
Examples of digitization of operating procedures in a biobank.

Even before collections begin, digitization can streamline workflows (Figure 3).

Today, patient informed consent is often still obtained in hard copy. The use of an electronic consent form optimizes the workflow and can be stored directly in the clinical information management system [13]. It is relevant to develop digital consent management platforms that will allow patients to edit their personal and/or contact details and their consent decisions, as well as track the results of the studies [14,15].

Automated stations for biospecimen transportation and aliquoting of biological samples, as well as automated freezing/storage systems, can be used for this purpose [10]. It enables digital tracking of storage conditions of a biospecimen and its subsequent routing.

Furthermore, biomaterial collection tubes with barcodes or QR codes are widely used today. Registering these tubes in an electronic system will ensure that the biomaterial is linked to the donor’s ID, thus reducing the risk of confusion [7]. Radio frequency identification (RFID) technology can be an alternative to barcoded tubes. RFID labels have several advantages, namely:

- They contain more information and do not require visualization for reading;
- They can be placed inside a transport box or a cryotube as well as inside liquid or tissue samples, thus enabling monitoring of temperature during storage, transportation or during the pre-analytical stage [16].

Real-time digital temperature monitoring is an essential tool in biobank organization [17] since temperature fluctuations during both the pre-analytical phase and storage may deteriorate specimen quality by affecting various metabolites [18,19]. Today, such digital tools are often already integrated into modern low-temperature freezers and liquid nitrogen storage systems.

Promising trends involve launching and using augmented reality glasses to facilitate biospecimen collection or track their transfers in a cryopreservation facility [13] and using drones to transport biomaterial from a biobank to medical facilities, which would allow these facilities to adapt to the lack of transport infrastructure (e.g., in rural areas or in the field) and minimize transportation time [20].

Digital technologies can also be helpful for biobanks storing collections of formalin-fixed, paraffin-embedded (FFPE) tissue sections. FFPE tissue sections that are fully digitized as whole slide images can be stored in an image database together with annotations and pathology information. These digital images can subsequently be analyzed by employing artificial intelligence or machine learning for research purposes [21,22,23].

### 3.2. Comprehensive Biobank Information Management System

Information stored in a biobank database can usually be subdivided into several types: patient’s (donor’s) clinical data, demographic data, characteristics of biomaterial specimens, and administrative data [24]. When creating a biobank database, clinical data are usually manually extracted from patients’ medical records and questionnaires, and enormous intellectual and physical resources are used to build a specific database architecture each time a new research project is initiated [25]. As a result, because there are no uniform standards in the organization of clinical or other related data in the biobank database architecture, challenges arise related to data selection and pooling, limiting the collaboration possibilities and preventing the full-scale use of biobank resources [26,27].

Digitalization will allow modern biobanks to perform digital collection and pooling of different types of data [23]. Biobank information management systems specifically adapted to biobank workflows are among the essential technological requirements of a modern biobank. Transparency and traceability of activities are achieved by documenting each step related to biospecimen processing. Many management systems offer options for storing data by annotating the biospecimen and merging several data sets (patient’s lifestyle, anthropometric data, anamnestic data, as well as clinical and “omics” data) into a single searchable digital platform [28].

Omics data (genomics, transcriptomics, proteomics and metabolomics) provide the opportunity to comprehensively study biological processes in different states of human health and disease. Such data allow us to identify new molecular interactions both within one protein, transcript, and metabolite layer and between layers [29]. However, genomic and omics data require large amounts of memory. To cope with such a volume of information, biobanks are forced to implement complex data management systems that allow not only to store but also to effectively organize access to these data for researchers. The combination of omics data and donor metadata allows for a more accurate interpretation of results and improves the research quality [30].

The following data management systems are currently being used intensely in healthcare:

- The Laboratory Information Management System, which is utilized for processing data related to the lifecycle of a biospecimen;
- Hospital information systems for monitoring patient data;
- The system that monitors and controls the temperature and levels of liquid nitrogen. Biobank information management systems can also interact with these software systems and databases. Thus, biobank information management systems are often the subsets of the Laboratory Information Management System repurposed and customized to meet the needs of a biobank [12].

Linking biobanks into a data-sharing platform is a powerful tool for establishing communication between biobanks and researchers. This communication will facilitate searching for biomaterial and associated data to conduct high-quality research. It is promising to develop a direction that would enable nationwide or international integration of different IT tools, which in turn will improve the level and quality of research in different areas of biomedicine [31,32]. Educational programs aimed at training medical staff in basic aspects of biobanking can be empowered and implemented by using digital platforms. This educational process is important due to the fact that it is the medical staff that obtains the patient’s informed consent, collects and annotates biomaterial, and participates in the pre-analytical preparation of biospecimens [33].

## 4. Digital Biobanks as a Part of the Global Biobanking Market

The establishment of digital biobanks is an innovative solution to overcome the problems associated with traditional biobanks, namely their closed nature and limited resources for storing and structuring large datasets. By harnessing the potential of technology, digital biobanks offer easier access and efficient data retrieval. Digital biobanks provide tools for analyzing data and identifying correlations between biomaterials and clinical parameters. In addition, the feasibility of communication between biospecimen holders and interested researchers through the digital biobank platform opens up new ways for establishing cooperation and new collaborations in research; the use of a single centralized database allows data tracking, quality control and improved circulation, thus revolutionizing the biobanking industry [32,34].

Obtaining specific biological specimens for research purposes has conventionally been a challenging and time-consuming process that often requires complicated logistics and significant resources. However, digital biobanks can simplify and optimize this process, making procurement of required specimens easier than ever before. Due to secure online platforms and carefully organized databases, researchers can now search across a vast collection of well-annotated specimens associated with comprehensive clinical and molecular data. This dataset will allow researchers to make better-informed decisions, choose proper specimens for their research, and speed up scientific discoveries [35,36].

The intense development of digital biobanking predicts that the number of users will increase significantly since these services are highly demanded in the industry [32]. To meet the demands of the growing number of users, the market will increasingly focus on the improvement of digital biobanks. This customer-centric approach will ensure that the needs and requirements of all the stakeholders are effectively met, thus contributing to further growth of the biobanking market in general and the increasing importance of digital biobanks in particular; as a result, this phenomenon will fit harmoniously into the evolving biobanking industry [37,38]. Table 1 summarizes the currently available digital biobanks and their characteristics.

Collaboration between the ProMPT and Southern Collaborative projects has yielded the ***UK Post Cancer Sample Collection Database***, which involves an extensive collection of human biospecimens (DNA, RNA, urine, fresh tissue samples, and FFPE samples). The database is available for the scientific community and allows for the request of biospecimens for research projects.

***DxConnect Virtual Biobank*** offers biobank stakeholders the opportunity to register their biobank or a biobank network. The platform allows users to search for biobanks or a biospecimen sample using a keyword list. However, it may take at least 6 months to provide some data for the selected specimens. Furthermore, there is also no customized registration system for users who are not biobank stakeholders. It is also worth noting that the most recent updates to the platform were made back in 2021, which may indirectly attest to poor community interest or inefficient promotion of the platform.

The Australian Partnership for Preparedness Research on Infectious Disease Emergencies (APPRISE) consortium and the BioGRID company have developed ***APPRISE Virtual Biobank****,* a virtual biobank for empowering research on infectious diseases in Australia. APPRISE acted as an expert in infectious diseases, while BioGRID designed the technical architecture. The APPRISE Virtual Biobank provides access to the existing collections of biospecimens of infectious diseases, including several biomaterial types (plasma, serum and peripheral blood mononuclear cells). An advantage of the solution offered by BioGRID is that researchers can easily use a single web platform to access a catalog of biospecimens from several collections. The data stored in separate collections are neither “moved” nor transferred to the central database: data aggregation for the platform is performed with preserved individual control and access mechanisms. The portal is easily scalable for integrating additional collections.

BBMRI-ERIC and UK Biobank, large well-proven biobanking organizations, also have web portals for searching for biospecimens and associated data.

The ***BBMRI-ERIC*** catalog is a tool that collects and distributes information about biobanks across Europe that are willing to share their data and/or specimens as well as collaborate with other research groups. The catalog offers tools to search for biospecimens and data for researchers, while for biobanks, it provides a platform to exchange information about the existence of assets and services, as well as communicate with researchers interested in them.

***UK Biobank*** has collected and continues to add data to its biocollection. Since 2006, the donors of UK Biobank have provided a very broad range of data about their health, physical activity, impacts of various factors (e.g., diet and eating habits), mental health, as well as the omics and diagnostic data. An online community has been established for UK Biobank users. Hence, UK Biobank Data Showcase provides access to information about 500,000 donors and contains not only the reference information on how these data were collected but also notes on future collections.

***German Biobank Node*** and German Biobank Alliance have created an AI network that aggregates biobanks both within the Consortium and international-level ones (such as BBMRI-ERIC). Sample Locator is a tool that allows researchers to search for biospecimens and associated data in biobanks, which are mostly located in Germany. To request specimens, users need to be authorized through their institutional ID or via LifeScience Hostel. The authorized users can select target biobanks, and the data about their availability will be automatically transferred to the Negotiator platform [48]. The developed infrastructure enhances the visibility of biobanks and empowers biomedical research.

The ***BioIVT*** resource offers an opportunity to procure biospecimens on a commercial basis. The product range involves blood derivatives (bone marrow, buffy coat, umbilical cord blood, leukopak, menstrual blood, plasma, serum, platelets, red blood cells, and whole blood), cellular and molecular (DNA, RNA) products, tissues, etc. Along with biospecimens, BioIVT offers customized high-quality solutions such as in vivo metabolite identification, highly sensitive quantification of medicinal products and/or their metabolites, bioanalysis of oligonucleotides in various biomatrices, toxicokinetic analysis, etc. Thus, BioIVT is an independent commercial resource where the user can purchase a product and/or a service, which makes it similar to a marketplace.

Another commercial project, the ground-breaking ***iSpecimen Marketplace****^®^,* offers biospecimens (biofluids, tissue samples, stem and immune cells, DNA- and RNA-sequenced cancer tissue) for purchase. The unified interface of iSpecimen allows researchers to access a network of hospitals, biorepositories, practical groups, commercial laboratories, medical facilities and other healthcare organizations that provide access to millions of thoroughly annotated biospecimens and patient data. Orders can be tracked and managed in a personal account.

The ***BioVitrina*** web repository is a similar product; unlike iSpecimen, it is noncommercial. BioVitrina has been developed to optimize the infrastructure of biobanks for organizations and make the information about bioresource collections stored in them available. Biospecimens in the catalog are systematized in accordance with the International Classification of Diseases 10th Revision. After choosing the biospecimens most suitable for a specific research problem, the user can make a request to receive them. A prerequisite for obtaining biospecimens is that the results of experimental studies will be subsequently published in the BioVitrina web repository. This exchange of analytical results of studies will help avoid repeating similar studies and contribute to material resource conservation.

Digital biobanks, especially if coordinated by national and international legislation, can be an indispensable resource for advancing academic research, the pharmaceutical industry and translational research as well as speeding up preclinical and clinical research. Having an enormous potential to fulfill the demand for biospecimens among users, virtual databanks allow researchers to obtain a sample of biospecimens they are interested in relatively easily [49].

## 5. Problems and Limitations

Along with advances in digitalization in biobanking, various issues related to data confidentiality and security become topical. It is important to integrate the tools for data management and use into the IT infrastructure in compliance with legal and ethical regulations.

Despite the multiple optimistic views of the potential of the evolution of biobank digitalization, some issues require special attention (Table 2).

### 5.1. Data Confidentiality and Security

The importance of security protocols and compliance with privacy principles for large datasets stored in biobanks are among the most important and top-priority factors that need to be carefully considered when discussing biobanks, in general, and digital biobanks, in particular [55,56].

Due to the application of modern encryption and access management tools, digital biobanks are protected against any potential security breaches and simultaneously guarantee that only authorized personnel can access sensitive information [56,57]. Moreover, strict and elaborate internal data management rules and protocols control vast and complex datasets, ensuring accurate and careful use of these data [58]. The security and privacy of personal data are further strengthened using advanced data anonymization techniques ensuring that any identifying attributes were removed and that the data were completely de-identified [55]. The application of differential privacy and federated learning technologies (which are employed in machine learning-assisted research), as well as various data protection protocols (Table 3), allow one to perform complex and collaborative research. while maintaining the confidentiality of individual subjects [58,59,60].

It is also important to use transparent and complete procedures for informing donors and obtaining consent, which ensure that participants are fully informed about the objectives and potential risks associated with the use of their data and can significantly increase trust and guarantee that their rights are protected [61].

### 5.2. Standardization of Specimen Collection and Storage

Specimens whose associated information is stored in digital biobanks should be collected and stored in compliance with the generally accepted standards in order to ensure reliability and uniformity between different digital biobanks. International organizations such as ISO have set common standards for biospecimen storage and transportation [61,62]. Such parameters as temperature maintenance, minimal exposure to contaminants, and specimen retrieval standards must be followed to preserve specimen integrity and quality [61,63].

Automated biospecimen monitoring systems that can track and validate biospecimens according to specified parameters help enhance quality control. Thus, LIMS products for monitoring laboratory equipment are such systems. They provide continuous monitoring, ensuring that any deviation from the set standards is detected and eliminated in a timely manner [62,64].

### 5.3. Ethical and Legal Aspects

Although there are obvious advantages in using digital biobanks as well as web technologies for making the existing biobanking structure more efficient, accessible and robust, it is extremely important that ethical considerations are paramount and are taken into account when developing this industry [61]. Since biobanking is currently undergoing a transformation towards digitalization and virtualization, it entails many potential risks and ethical issues that need to be carefully analyzed, assessed and taken into consideration [65,66].

Research involving human subjects raises many ethical issues, ranging from obtaining consent, privacy and confidentiality, data security, honesty, transparency and potential stigmatization, to appropriate use of stored specimens and data, protection of rights and welfare of human subjects, as well as managing potential risks of discrimination, exploitation and abuse [66,67,68].

Along with obtaining informed consent, donors should have a right to withhold their consent and have their specimens and data removed from any biobank. Informed consent should be well-drafted and touch upon all the possible aspects, including comprehensive information about the goal of biobank functioning, as well as ethical aspects of the experiments conducted using biospecimens collected from the donors [69,70].

The following criteria should be used to assess the ethical aspects of modern biobanks:

- Obtaining informed consent from donors and providing them with comprehensive information and a right to withdraw their biological material at any time;
- Ensuring the security of the data stored in digital biobanks to prevent risks of jeopardizing privacy, personal data security, and potential discrimination of donors;
- Ensuring transparency of biobank management and explicit rules for involvement of commercial subjects in research;
- Using the biospecimens exclusively to conduct ethical experiments that aim to benefit humans and society.

Since digital biobanks have emerged relatively recently, it is difficult to assess how well legal regulations in this industry have been established. However, the General Data Protection Regulation (GDPR) in the European Union is a prime example of legislation that addresses ethical issues in this industry. The GDPR has been adopted by the European Union to improve and modernize the rights of European citizens with respect to data protection and confidentiality. It also addresses the export of personal data outside the European Union. One of its aims is to give individuals better control over their personal data. The GDPR significantly affects biobanks storing personal data of European citizens’ as it establishes a requirement to obtain explicit consent to store data and gives individuals rights to access, edit and delete their data. It is rather challenging to implement the GDPR in biobanks, especially in the case of international data sharing, and more stringent procedures for obtaining consent are required. The GDPR also enforces data subjects’ rights and the accountability of data controllers, foreseeing significant penalties for non-compliance. There should be a balance between allowing data to be used in ethically approved studies and the protection of human rights. Compliance with the GDPR can help increase trust in data use [71,72,73]. The Genetic Information Nondiscrimination Act (GINA), adopted in 2008 in the USA to protect individuals against the misuse of genetic information by employers and health insurance providers, is another example of the law regulating the ethical aspects of biobanks. This law prohibits the use of genetic information in employment decisions, makes it illegal for employers to request or use genetic information to make hiring, firing, or promotion decisions, and if such cases do occur, allows affected employees to take legal recourse, as well as prohibits the use of genetic information when determining insurance premium or coverage [74,75]. Studying the regulatory frameworks of different countries in this industry helps with making laws in countries where these aspects are either not regulated at all or regulated insufficiently (Table 4).

In their study, Shickle et al. aimed to highlight some challenges faced by biobanks and proposed a classification of biobank networks according to the following categories: (1) storage networks, (2) bring-and-share storage networks, (3) catalog networks, (4) partnership networks, (5) contribution networks, and (6) expertise networks, thus demonstrating that many networks may have functions falling into several categories because of their activity range [76].

## 6. Conclusions

Well-characterized large biospecimen samples are demanded in studies aiming to develop novel biomarker detection methods as well as targeted diagnosis and treatment strategies [2]. Small patient samples are also currently recruited to participate in trials in isolated study sites. Establishing biobank networks that employ the same standards of biospecimen collection, data analysis and storage, annotation structuring, followed by pooling these data using an accessible digital platform will make it possible to provide custom-size samples upon request more promptly, efficiently and at a lower cost to attain statistically significant results in research. Data digitalization and establishing a common database allows one to generate a web catalog of specimens characterized in terms of their clinical and anthropometric parameters that can be accessed by authorized users [77].

Digital biobanks are ideally suited for large-scale studies conducted under nationwide and international projects. Furthermore, they can also be demanded by individual research groups partnering with companies that foster a specific industry (biopharmaceutical, agricultural, etc.).

It is relevant to integrate biobank information control systems with medical and laboratory information systems to acquire data on stored specimens, their donors, as well as the list and the results of laboratory studies. This scalable IT solution suits biobanks controlled by the government, commercial enterprises, or private research institutions working with all types of specimens.

## Figures and Tables

**Table 1:** Examples of current digital biobanks.

Name of Digital Biobank	Description	Types of Biomaterials	Dataset Size	Territory Covered	Research Type	Data Availability	Data Access Format	International Compliance	Specimen Request	Specialty	Access
UK Post-Cancer Sample Collection Database [39]	The first virtual biobank devoted to prostate cancer research	Tissues, blood, DNA	>10,000 biological specimens	UK	Oncology	On request	Web portal, API	Yes	Yes (available for researchers residing in UK)	Prostate cancer	Free of charge
DxConnect Virtual Biobank [40]	A platform providing access to clinical specimens for researchers. It allows one look through collections registered by any institution worldwide	Different types of clinical specimens	N/A	Global	Various	On request	Web portal	N/A	Yes	Multi	Free of charge
APPRISE Virtual Biobank [41]	Virtual biobank with information about specimens collected from patients with an infectious pathology	Plasma, serum, peripheral hematopoietic stem cells	58,000 specimens from 2700 patients	Australia	Infectious diseases	Yes	Web portal	N/A	Yes	Infectious diseases	Free of charge
BioIVT [42]	A platform offering access to an extensive repository of biological specimens and clinical database for research purposes	Different types of biological specimens	>1,000,000	Global	Various	Yes	Web portal	Yes	Yes	Multi	On a fee basis
BioVitrina [43]	A web-repository offering access to information about well-annotated biological specimens of various pathologies	Tissues, urine, blood serum, blood Plasma	>1000	Russia	Various	Yes	Web portal	Yes	Yes	Multi	Free of charge
BBMRI-ERIC [44]	The European infrastructure of biobanks offering access to bioresources and services to support biomedical research	Different types of biological specimens	>1520 collections of biological specimens	European Union	Medical research, genomics	Yes	Web platform	Yes	Yes	Biomedical research	Depends on a biobank
UK Biobank [45]	One of the largest and most comprehensive biobanks in the world, which provides data and specimens for conducting research in the field of genetics and population health	Genetic data, clinical data, blood samples	>500,000	UK	Various	Yes	Web platform	Yes	No	Health and diseases	Free of charge
iSpecimen Marketplace [46]	iSpecimen serves as a centralized platform for sourcing high-quality human biospecimens from a global network of providers.	Different types of biological specimens	>1,000,000	Global	Various	Yes	Web platform	Yes	Yes	Multi	
German Biobank Node [47]	Serves as a central platform for collaboration among German biobanks and represents their interests within the European BBMRI-ERIC network. It aims to standardize quality management and create an IT infrastructure for accessibility to biomaterials and related data for biomedical research	Different types of biological specimens	N/A *	European Union	Various	Yes	Web platform	Yes	Yes	Precision medicine	

* German Biobank Node unites 36 academic biobanks and one IT center.

**Table 2:** Certain issues related to the evolution of biobank digitalization.

Problem	Description	Solution
Big data *	Researchers’ demand for accessibility of “broader” data requires the acquisition and administration of large biospecimen collections and associated data, often from several sources	Paradigm shift both in requirements for storage and in data analysis. The use of computerized mechanisms, implementation of various solutions: from cloud storage to creating secure dedicated repositories
High speed of data acquisition	Due to the development of “omics” technologies (proteomics, genomics, etc.), new information about biospecimens appears at a fast pace	
Standardization of analytical data	Variations related to collection, processing, and storage of different specimens and associated clinical data significantly hamper extrapolation or pooling data obtained in different studies	The use of unified terminology and international best practices [50]
Ethical problems	For collecting specimens and data, personal data that are often identifiable need to be accessed. Sometimes donors cannot fully assess the type or focus of a study conducted due to the lack of knowledge (expertise) or it may be necessary to send study results to the donors, which in turn may cause technical challenges when drafting informed consent	Adaptation to new legal acts (e.g., the EU General Data Protection Regulation (GDPR)) [51]
Separation of biobanks	Organizations at which biobanks have been founded and that fund their infrastructure often assume that biobanks must become “independent” at some point. However, most often it cannot be attained, especially for the biobanks integrated into research or medical institutions [52]	New, more flexible funding models are needed, which will empower the development of biobanking, thus unlocking the potential of opportunities emerging along with advances in precision medical research
Data integration	Integrating data from multiple studies becomes challenging due to differences in formats, data structures, and methodologies used across studies and platforms	Network-based data integration tools (OmicsNet [53], etc.) allow combining data from different studies. MetaboAnalyst [54] can perform cross-platform analyses by aligning metabolomic data with proteomic or transcriptomic data

* “Big data” is a blanket term describing the use of novel computing technologies and software to retrieve knowledge from extremely large and heterogeneous data sets such as biological and medical data.

**Table 3:** Methods for ensuring data security.

Data Protection Technique	Protocols Used	Description
Data encryption	SSL/TLS, AES, RSA, SSH, PGP/GPG	Data encryption is used to protect the confidentiality of data by transforming them to a format that is incomprehensible to third parties. Examples of encryption algorithms include SSL/TLS for secure data transmission over the network; AES and RSA for protecting data at rest; and SSH for providing secure remote access to systems.
Authentication and authorization	LDAP, Kerberos, OAuth, OpenID Connect	Authentication is the process of verifying the identity of a user or a device. Authorization is determining whether a user has rights to access resources after successful authentication. Examples of protocols include LDAP for centralized account management; Kerberos for secure authentication; and OAuth and OpenID Connect for authorization and authentication in web applications.
Access control	RBAC, ABAC, ACL	Access control determines which users have access to which resources. Examples include RBAC (Role-Based Access Control), where access depends on the user’s role; ABAC (Attribute-Based Access Control), where access depends on the user’s attributes; and ACL (Access Control Lists), where access to resources is set up manually.
Monitoring and audit	SIEM, SNMP, Syslog	Monitoring and audit are used to track user activity and detect potential security threats. Examples include SIEM (Security Information and Event Management) for analyzing and aggregating event logs, SNMP (Simple Network Management Protocol) for monitoring network devices, and Syslog for centralized logging of events.

**Table 4:** Legal norms regulating the ethical aspects and data storage rules in the biobanking industry in different countries.

Country	Law	Description	Ethical Issue to Be Solved
European Union	GDPR (General Data Protection Regulation)	Controls personal data processing and storage; requires consent from data subjects for data processing; confers rights to data subjects.	Personal data protection and enforcement of rights for maintaining data confidentiality.
USA	GINA (Genetic Information Discrimination Act)	Prohibits discrimination based on their genetic information in health coverage and in employment.	Prevention of discrimination based on one’s genetic information, protection of individuals’ rights.
Japan	The Law on Human Genome and Biobanks	Regulates the management and use of genetic data; measures to protect personal data of donors.	Regulation of using genetic data, protection of donors’ personal data, management of the access to biological specimens.
China	National Ethical Guidelines for Biobanks	Sets standards for collection, storage, and use of specimens, as well as requirements for obtaining consent and confidentiality.	Setting standards for protecting donor confidentiality, regulation of obtaining consent for specimen use.
Germany	Law on Data Protection and Bioethics	Regulates handling personal data in medical research, including obtaining consent for the use of biological specimens.	Protection of personal data in medical research, enforcement of confidentiality rights.
UK	Human Tissue Act	Regulates the use of human tissues and cells by setting requirements for obtaining consent and using specimens for research purposes.	Properly obtaining consent for using human tissues and cells; regulating access to specimens for research purposes.
Russia	Federal Law “On Personal Data”	Regulates handling personal data, including the data collected during medical studies and biobanking, ensuring personal data protection.	Protection of personal data, including the medical and biological data; prevention of data misuse.

## Data Availability

This is a review paper that collected data from public sources listed in the “References” and from the open-access web-source PubMed.
